# Supplementary material for: Patterns of Intron Gain and Loss in Fungi
Source: PLoS Biol. 2004 Nov 30;2(12):e422. doi: 10.1371/journal.pbio.0020422 (PMC532390; doi:10.1371/journal.pbio.0020422)
Supplement: Table S1 — Also available at http://genes.mit.edu/NielsenEtAl/. (4.3 MB ZIP). [file pbio.0020422.st001.zip › NielsenEtAl/html/1268.html]

AN4566.1.NCU04251.1.MG09551.1.FG10327.1


```
 CLUSTAL W (1.82) Multiple Sequence Alignments - Introns Inserted


Sequence 1: MG09551.1	890 aa
Sequence 2: FG10327.1	898 aa
Sequence 3: NCU04251.1	888 aa
Sequence 4: AN4566.1	983 aa
Alignment Length: 1012 aa
Number Identitical Residues: 487 aa
Alignment Score (without introns) 23323


MG09551.1 	-------~-------------MNNRP-----PPHRTPSPGHPLQHGYQLDDAPY--GRPG
NCU04251.1	-------~-------------MDPRM-------HTAPPAGHPIQPGYQLEDNPY------
FG10327.1 	-------~-------------MDPRYGAQPMPPRRSPSPGHPVQPGYQLDDNPFDDGRYG
AN4566.1  	MSYNRLG1DPYGDDRDARSPIMNPSS-----LSNRSPSPGRPLD-GYQLSDAPYG-----
          	 : .  . .. ... .: :. *:         .. :*..*:*:: ****.* *:.     

MG09551.1 	LNSASNLDIPMG----PGRH-TPSD~QLQLHTA0HSMANMSEPGYQQQPGGYR-DEYSVN
NCU04251.1	-HHQQGFDIPAG----PGRY-SPGD~ALHIQTP0QPIEGMG--GYNAPGQHYT-PDYAVN
FG10327.1 	QYGPSSQHLAMPGHDPHGRLPTPSD~HLSLNAA0QSVDNLA--GYGPPSGG----DYAIN
AN4566.1  	--HHHHIEMPSS-----DRLAEQPT0YSVERIP~QSYGHNE--AYEAQHQHYPGYEYSVD
          	       .:.       .* .         . . :.       .*        . :*:::

MG09551.1 	PEQHHDAYYNPTYTP--TPNEAQTPYG----EPGYEH---DGRPLLPQ---QDSYGQYSD
NCU04251.1	PEEHHDAYYNQPYEP--QVGHDPYAAAPTPPVAGYQAHD-DQRPMLMH---TDSQVGQSD
FG10327.1 	PEAHHDAYYNQPYEP--RPQHQGYDQGY---EQDYDVP--DNRPMLPH---QHS-DVPSE
AN4566.1  	PEAHHDAYYTQPYQPTVTPGHDDYDLGQ---YPGHQHSYQDDEPILQPEDPFQAQNPYSD
          	** ******. .* *:    .     .      .::   .* .*:*  ... .:    *:

MG09551.1 	NPQQQQQ-----QQGGLKRWKTVKRVPLYMGNLVLDCPVPPKLLNQFPHGE---RDEFTH
NCU04251.1	PYHDEPQPP--TNNAPIKRWKTVKQVLLYRGNLVLDCPIPPKLLNQLPHGE---RDEFTH
FG10327.1 	QYQDPPQ-----QGGGIQRRKTVKQFALYRGNLVFDCRVPPLLLQQNPHGE---RDEFTH
AN4566.1  	DYQEDMTIAPTPSPAPLRRWKTVKEVQLFQGNLVLDCPIAPKLLNQIPHAENGQRDEFTH
          	  ::    ..:.. . ::* ****.. *: ****:** :.* **:* **.*...******

MG09551.1 	MRYSAATCDPNDFYDEKFTLRQRLFSKPRETELFIVVTMYNEDEVLFARTMIGVFKNIEY
NCU04251.1	MRYSAATCDPSEFYEENFTLRQKLFSKPRHTELFIVVTMYNEDEILFARTMIGVFKNIEY
FG10327.1 	MRYSAATCDPNDFHDHDFTLRQRLFSKPRHTELFIVVTMYNEDEILFARTMTGVFKNIEY
AN4566.1  	MRYSAATCDPKDFFEERFTLRQKLFAKPRHTELFIVVTMYNEDDFLFARTMVGVFKNIEH
          	**********.:*.:. *****:**:***.*************:.****** *******:

MG09551.1 	MCKRPESKTWGKEAWKKIVVCIVSDGRAKINPRTRALLAGLGVYQEGIARQNVDDKPTTA
NCU04251.1	MCKRTESKTWGKDAWKKIVVCVVSDGRAKINPRTRALLAGMGVYQEGIAKQQVNGKDVTA
FG10327.1 	MCNRPNSKTWGKDAWKKIVVCVVSDGRSKINPRTRALLAGMGVYQEGIAKQHVNDKAVTA
AN4566.1  	MCSRTRSKTWGKDAWKKIVVCVISDGRAKINPRTRAVLAGLGCYQDGIAKQQVNGKDVTA
          	**.*..******:********::****:********:***:* **:***:*:*:.* .**

MG09551.1 	HIYEYTTQIGMALKNDVVQLLPRQ-QPVQLLFCLKENNQKKINSHRWFFSAFGRVLNPNI
NCU04251.1	HIYEYTTQVGMTIKNDVVQLIPKQ-QPVQMLFCLKEKNQKKINSHRWFFQAFGRVLDPNI
FG10327.1 	HIYEYTTQTHLQIKNNVVQLVHRR-QPVQMLFCLKEKNAKKINSHRWFFNAFGRVLDPNI
AN4566.1  	HIYEYTTQVGMELKGNQVHLKPRSGVPVQMIFCLKEKNQKKINSHRWFFQAFGRVLDPNI
          	********  : :*.: *:*  : . ***::*****:* **********.******:***

MG09551.1 	CVLLDAGTKPGGNSIYHLWKAFDLEPMCAGACGEIKAMLG1TGGKNLFNPLVAAQNFEYK
NCU04251.1	CVLIDAGTKPGGSSIYHLWKAFDLEPMCAGACGEIKAMLG~TGGKNLINPLVATQNFEYK
FG10327.1 	CVLLDAGTRPGGSSIYHLWKAFDLEPMCGGACGEIKAMLG~TGGRYLLNPLVAAQNFEYK
AN4566.1  	CVLLDAGTQPGKDSIYRLWKAFDVEPMCGGACGEIKVMLD~HG-KKLFNPLVAGQNFEYK
          	***:****:** .***:******:****.*******.**.  * : *:***** ******

MG09551.1 	MSNILDKPLESAFGFISVLPGAFSAYRYIALQNDKNGQGPLEKYFAGEKLHGAG-AGIFT
NCU04251.1	MSNILDKPLESAFGFISVLPGAFSAYRYVALQNDKNGQGPLEKYFAGEKLHGGD-AGIFT
FG10327.1 	MSNILDKPLESAFGFISVLPGAFSAYRYVALQNDKNGKGPLEKYFLGETLHGGSEAGLFE
AN4566.1  	LSNILDKPLESAFGFISVLPGAFSAYRYIALQNDKNGQGPLERYFLGEKMHGAN-AGIFT
          	:***************************:********:****:** **.:**.. **:* 

MG09551.1 	ANMYLAEDRILCFELVTKRNCHWILQYVKSATGETDVPDTATELILQRRRWLNGSFFAAI
NCU04251.1	ANMYLAEDRILCFELVTKRNCHWILQYVKSATGETDVPADLTELILQRRRWLNGSFFAAI
FG10327.1 	SNMYLAEDRILCFELVTKRNCHWILQYVKSATGETDVPDTVTELVLQRRRWLNGSFFAAI
AN4566.1  	ANMYLAEDRILCFEIVTKRNCRWLLQYVKSSTGETDVPDQMAEFILQRRRWLNGSFFAAV
          	:*************:******:*:******:*******   :*::**************:

MG09551.1 	YAIAHFYQFFRSDHSFFRKIAFFIEFTFNTVNMIFAWFAI~GNFFLVFKILTTSLGDETL
NCU04251.1	YAIVHFHQFFRSDHSFLRKIAFFIEFVFQTVNMIFAWFAI~GNFFLVFKILTTGLGDEKL
FG10327.1 	YAIVHFLDFLRSDHTFLRKFAFFIEFIFNTINMIFAWFAI~GNFFLVFKILTTSLGDDTL
AN4566.1  	YAITHFYQLWRSDHSFIRKFMLLIETIYQTINMLFAWFGI0GNFFLVFHILTTYLGDADL
          	***.** :: ****:*:**: ::**  ::*:**:****.* *******:**** ***  *

MG09551.1 	LGTTGKILGVCFEWLYGVSLITCFVLAMGNRPAGSGPYYLAMIYFWAIIFC2YLLFAAVF
NCU04251.1	LGTVGQILGVVFAWAYGVTLITCFVLSMGNRPAGSPRLYMGMVVFWAIIFI2YLMFAAIY
FG10327.1 	LGRTGEILGVVFTWLYGVFLITCFVLSLGNRPAGSGRLYTAMCWFWAIIMI2YLMFAAVF
AN4566.1  	LGTAGKVLGVVFEWLYLATLVTCFVLSLGNRPGGSNKLYMTMVYLWVFIMI~YLAFAAVF
          	** .*::*** * * * . *:*****::****.**   *  *  :*.:*:  ** ***::

MG09551.1 	ISVKAIMADAQR--GLSVPELLKDQVVVTLILSIMSTYGIWLVASLLMFDPWHMFTS~LV
NCU04251.1	IAVVAIQTDVQK--GLSFTDLFRNELFYTLIVSVVSTYGIWLIASLLMFDPWHMVTS~MV
FG10327.1 	ISVKAIIADVNDENGFNISDIFKNKVFYMLIISLMSTYGIWLIASLIMLDPWHMVTS~FA
AN4566.1  	VTVRSIQEEVKD-GSFTFSTLFTNSTFFSIIVSLGSTYVMWFIASIIFMDPWHMFTC0FI
          	::* :*  :.:  ..:... :: :. .  :*:*: *** :*::**::::*****.*. : 

MG09551.1 	QYMLLSPTFTNVLNVYAFCNTHDISWGTKGDDKPDKLPSVSTK-DGAGKADLP-DEADLN
NCU04251.1	QYMLLSPTYTNVLNVYAFCNTHDISWGTKGDDKPDKLPSVNTK-DGQGKTDLP-DEGDLN
FG10327.1 	QYMLLTPTFTNVLNVYAFCNTHDVSWGTKGDDKVEELPSVNTK-DGTGKTDLP-DEGDLD
AN4566.1  	QYILLTPTYINVLNIYAFCNTHDITWGTKGDDKAEKLPSANLKPGGKVDVNIPQDDGDLN
          	**:**:**: ****:********::******** ::***.. *..*  ..::*.*:.**:

MG09551.1 	TMYERELAVFAQKHVEEKKALTPSQLDEKQLDYYRGVRTVVVLLWMVTNFGLAAVVLSTA
NCU04251.1	ASYERELQVFSRKYVKPVTAPTSAQLEEKQMDYYRGVRSMVVLVWMITNFALCAVVLSTA
FG10327.1 	AQYQREVAVFAQKFVEVKSAPTPSQLQERQMDYYRGVRTGVVLLWMVTNFGLAAIVLSSA
AN4566.1  	AQYEAELMKFAQKPPKEIKTISE---EERQADYYKGFRSSVVLVWVFCNFALGAVVLSSA
          	: *: *:  *::*  :  .: :    :*:* ***:*.*: ***:*:. **.* *:***:*

MG09551.1 	GLDRITPNT-NAETKEQ-RATIYMAVVLYSVAALSGFKFIGAMWFLVVP1CFTLSGAAS-
NCU04251.1	GLERIDPEEGSQEQQTTKRATIYMSVVLWSVAVLSGFKFVGACWFLVVR~MFRGV-----
FG10327.1 	GLDRISTKE-DKEAEQLSRSNIYMSIVLWSVAGLSAFKFIGAMWFLVVR~MFRGV-----
AN4566.1  	GLDRFSDDAEAAETDRNNRAMIYMAVVLWSVAGLSIFKFLGAMWFLVVR~MVSIFSRFIS
          	**:*:  .    * .  .*: ***::**:*** ** ***:** *****   .   .   :

MG09551.1 	------------------------------------------------------------
NCU04251.1	------------------------------------------------------------
FG10327.1 	------------------------------------------------------------
AN4566.1  	SVSTASNMICSSEVSNLFHRSRITLQAFNGRRFLSIPAGSAYRNDDKDNGLWNVIVFMSD
          	: ::::.  .::. :.    :  : .: ..    : .:.::  .......  .     :.

MG09551.1 	
FG10327.1 	
NCU04251.1	
AN4566.1  	
          	
```
